# Osteoporosis and Rheumatoid Arthritis: Mechanisms Underlying Osteoclast Differentiation and Activation or Factors Associated with Hip Fractures

**DOI:** 10.3390/jcm14041138

**Published:** 2025-02-10

**Authors:** Takeshi Miyamoto

**Affiliations:** Department of Orthopedic Surgery, Faculty of Life Sciences, Kumamoto University, 1-1-1 Honjo, Chuo-ku, Kumamoto 860-8556, Japan; miyamoto.takeshi@kuh.kumamoto-u.ac.jp; Tel.: +81-96-373-5226

**Keywords:** rheumatoid arthritis (RA), osteoporosis, chronic inflammation, glucocorticoid, aging

## Abstract

Osteoporosis is defined as a condition of increased risk of fracture due to decreased bone strength. In developed countries, the number of patients with osteoporosis and fragility fractures has been increasing in recent years due to the growing elderly population, posing a social challenge not only to fracture patients and their families but also to the social healthcare economy. Osteoporosis can be divided into two categories: primary osteoporosis caused by aging or menopause and secondary osteoporosis caused by metabolic or inflammatory diseases or drugs such as glucocorticoids. The majority of patients have primary osteoporosis, and the pathogenesis of postmenopausal osteoporosis and factors associated with fragility fractures in the elderly have been elucidated. On the other hand, rheumatoid arthritis (RA) is one of the causes of secondary osteoporosis. RA is a chronic inflammatory disease characterized by joint swelling and destruction. Most often, treatment focuses on suppressing these symptoms. However, physicians should be aware of the risk of osteoporosis in RA patients, because (1) RA is a chronic inflammatory disease, which itself can be a risk factor for osteoporosis; (2) glucocorticoids, which are sometimes administered to treat RA, can be a risk factor for osteoporosis; and (3) patients with RA are becoming older, and aging is an osteoporosis risk factor. A comprehensive understanding of the pathogenesis of osteoporosis and its fragility fractures requires elucidating the mechanisms underlying osteoclast activation, which drives their development. Furthermore, identifying the factors associated with fragility fractures is essential. This review summarizes the pathogenesis of osteoporosis, the factors associated with fragility fractures, and the associations between RA and osteoporosis development.

## 1. Introduction

Osteoporosis is defined as a condition of increased risk of fracture due to decreased bone strength. Bone mineral density is responsible for about 70% of bone strength and structural and material bone quality for the remaining 30%. Bone mineral density (BMD) can be quantitatively measured by dual energy X-Ray absorptiometry (DXA), and diagnostic reference values have been established. On the other hand, the remaining 30% is defined by structural bone quality, such as bone microstructure, and material quality, such as the extracellular matrix protein composition. Structural bone quality is measured by micro-CT, high-resolution peripheral quantitative CT (HR-pQCT), or trabecular bone score (TBS), while material quality is measured by urinary pentosidine, but no diagnostic criteria to start drug treatment have been established. Osteoporosis can be classified as either primary osteoporosis, which is caused by aging or menopause, or secondary osteoporosis, which is caused by factors such as metabolic disease, inflammatory diseases such as rheumatoid arthritis (RA), and some medications. Osteoporosis is one of the typical age-related diseases whose prevalence increases with aging. In a survey conducted from 2015–2016, the number of patients with osteoporosis diagnosed by decreased bone mineral density over 40 years of age was estimated to be 15.9 million in Japan (4.1 million men and 11.8 million women), and the prevalence of osteoporosis in different age groups shows a clear increase with increasing age [1]. In addition, the 15.9 million patients with osteoporosis from 2015–2016 is a significant increase from the 12.8 million (3.0 million men and 9.6 million women) from 2005–2007 [2]. The increase in the number of osteoporosis patients is thought to be due to the increase in the geriatric population [1].

RA has complications [3], among them, osteoporosis, which should not be overlooked as it is directly related to bone fractures and to the resulting decreases in activity level of daily living (ADL) and quality of life (QOL). Additionally, there are various risks associated with osteoporosis, including RA [4]. RA patients can develop various risks of developing osteoporosis, including elevated levels of inflammatory cytokines in the blood or joints, glucocorticoid use, and aging. Bone alterations in patients with rheumatoid arthritis (RA) are basically assessed by bone mineral density loss, existing fragility fractures, and the Fracture Risk Assessment tool (FRAX) developed by the WHO for patients with primary osteoporosis [5], but in RA patients, joint erosion and/or destruction/deformation are also critical indicators for the local activation of osteoclasts.

RA patients have been shown to have a higher risk of fragility fractures than non-RA patients. A cohort study showed that the incidence rate (IR) per 1000 person years (PY) with 95% confidence intervals (CI) for the first fracture in RA patients was 18.3 (15.7–21.2) and the incidence rate ratio (IRR) was 1.32 (1.10–1.60) [6]. In addition, RA is listed as a risk for osteoporosis in FRAX [5], and many reports indicate that RA is a risk for osteoporosis [7,8]. However, despite the fact that RA patients are at high risk of fractures, osteoporosis is currently not adequately treated [8]. Physicians should always be aware that RA carries the risk of osteoporosis and fragility fractures.

To understand the pathogenesis of the disease, it is first necessary to understand how physiological bone metabolism is regulated. These mechanisms and pathways are essential for the regulation of bone homeostasis, and their disruptions can lead directly to changes in bone mass. Understanding the regulatory mechanisms of bone homeostasis has led to the discoveries of the receptor activator of the nuclear factor kappa B ligand (RANKL) as a therapeutic target for osteoporosis and bone erosion, Sclerostin as a target for osteogenesis-promoting drugs, and the mechanism underlying postmenopausal osteoporosis development [9,10,11]. Furthermore, the elucidation of the mechanism by which inflammatory cytokines cause bone erosion suggests the need to regulate inflammatory cytokines and related cellular activities in order to suppress bone erosion. Knowing the mechanisms underlying the osteoclast activation that causes osteoporosis development and fragility fractures and the factors associated with hip fractures in patients who actually developed hip fractures are mandatory for developing a way to prevent their developments. In the development of fragility fractures such as hip fracture, various factors play a role in the loss of bone mineral density and bone strength, including osteoclast activation. However, there are few reviews that introduce physiological osteoclast differentiation as well as osteoclast differentiation and activation under inflammatory conditions such as RA in a unified manner. In addition, there are no reviews that include factors that are characteristic of patients who actually developed hip fractures. This review aims to discuss the various factors and mechanisms involved in osteoclast activation, which causes bone loss and joint erosion, and the factors associated with fractures in patients with fragility fractures.

This manuscript mainly overviews and focuses on the literature related to the pathogenesis of osteoporosis and the mechanisms underlying the development of osteoporosis and joint erosion in RA. To highlight the importance of understanding the pathogenesis of osteoporosis and joint erosion, clinical articles showing the clinical aspects of RA, osteoporosis, and fragility fracture patients or cohort studies, together with the molecular mechanisms underlying these clinical outcomes, are alternately discussed.

## 2. Regulation of Bone Homeostasis

Bone homeostasis is mainly regulated by two cell types: osteoclasts, which are responsible for bone resorption, and osteoblasts, which are responsible for bone formation. Bone is not a static tissue with no metabolism, but bone homeostasis is continuously and dynamically regulated by bone resorption by osteoclasts and bone formation by osteoblasts. When the activities of osteoclasts and osteoblasts are balanced, bone is in a state of dynamic equilibrium and bone mass is maintained at a constant level. However, with menopause and aging, osteoclast activity relatively exceeds osteoblast activity, resulting in bone loss and osteoporosis.

Osteoclasts are terminally differentiated cells of the monocyte–macrophage lineage derived from hematopoietic stem cells and are the unique cells in the body responsible for bone resorption. The discovery of the receptor activator of the nuclear factor kappa B ligand the (RANKL)-RANK system has rapidly advanced our understanding of the mechanisms underlying osteoclast differentiation [12,13,14,15]. RANKL is a member of the tumor necrosis factor alpha (TNFα) superfamily of cytokines [16], and the osteoprotegerin (OPG)/osteoclastogenesis inhibitory factor (OCIF), a decoy receptor for RANKL, masks RANKL under normal conditions and prevents osteoclast differentiation [17,18]. Denosumab, a RANKL-neutralizing antibody, is used as a therapeutic agent for osteoporosis [9], a giant cell tumor of bone; metastatic bone tumors; and other diseases in which osteoclast activity is increased, resulting in decreased bone density and bone destruction. In Japan, denosumab is also used to inhibit joint erosion in RA [19].

After binding to its receptor RANK, RANKL activates various signals in osteoclast progenitor cells to promote osteoclast differentiation. The transcription factor c-Fos is essential for osteoclast differentiation [20]. c-Fos-deficient mice completely lacked osteoclasts, and the mice developed osteopetrosis [20]. Downstream from c-Fos, a transcription factor identified as essential for osteoclast differentiation is a nuclear factor of the activated T cells 1 (NFATc1) [21]. NFATc1 binds to the transcriptional regulatory regions of various osteoclast-specific molecules such as Cathepsin K and directly regulates their expressions [21]. NFATc1 also drives osteoclast differentiation positively by upregulating NFATc1 expression through auto-amplification, which induces NFATc1’s own expression [22]. RANKL stimulation activates calcium signaling through spleen tyrosine kinase (Syk) and Phospholipase gamma (PLCγ) from the immunoreceptor’s tyrosine-based activation-motif (ITAM) signal FcRγ and the DNAX activation protein of 12 kDa (DAP12), resulting in the expression and activation of NFATc1 [23]. Administration of FK506, an inhibitor of calcineurin also used as a treatment for RA, has been shown to suppress osteoclast differentiation by the suppression of NFATc1 but also suppresses osteoblast activity, resulting in decreased bone density [24].

## 3. Multinucleation of Osteoclasts by a Cell–Cell Fusion

It has long been known that osteoclasts become multinucleated by the cell–cell fusion of mononuclear osteoclasts. It was formerly believed that the multinucleation of osteoclasts requires cells to increase in size by fusion in order to form structures such as ruffled boarders and sealing zones, which are considered essential for osteoclasts to exhibit bone resorption capacity. Although various molecules have been reported to be involved in the cell–cell fusion of osteoclasts, an analysis of dendritic cell-specific transmembrane protein (DC-STAMP)-deficient mice showed that DC-STAMP is essential for the multinucleation of osteoclasts by cell–cell fusion of mononuclear osteoclasts [25]. Although osteoclasts from DC-STAMP-deficient mice were mononuclear, the expression of differentiation markers was similar to that of multinuclear osteoclasts from wild-type mice, indicating that DC-STAMP functions specifically for cell–cell fusion rather than differentiation [25]. Subsequently, the cell–cell fusion of osteoclasts was also demonstrated to be strongly inhibited in mice lacking v-ATPase V0 subunit d2 (Atp6v0d2), one of the subunits of the proton pump, and CD200-deficient mice [26,27]. Furthermore, the cell–cell fusion of osteoclasts was demonstrated to be completely inhibited in osteoclast stimulatory transmembrane protein (OC-STAMP)-deficient mice without affecting osteoclast differentiation [28]. An analysis of mice in which the cell–cell fusion of osteoclasts was inhibited has shown that although the cell–cell fusion of osteoclasts is not essential for resorption, it does increase its efficiency [25,28]. DC-STAMP and OC-STAMP were also shown to be essential for the cell–cell fusion of foreign-body giant cells (FBGCs), which are differentiated from the progenitor cells common to osteoclasts and become multinucleated by the cell–cell fusion of mononuclear FBGCs [25,28]. FBGCs are formed in artificial joints and other medical devices that are placed in the body. Both DC-STAMP and OC-STAMP have also been shown to be transcriptional targets of NFATc1 in osteoclasts [28,29].

## 4. Regulation of Osteoclast Differentiation by Transcriptional Repressors

**Figure 1:**
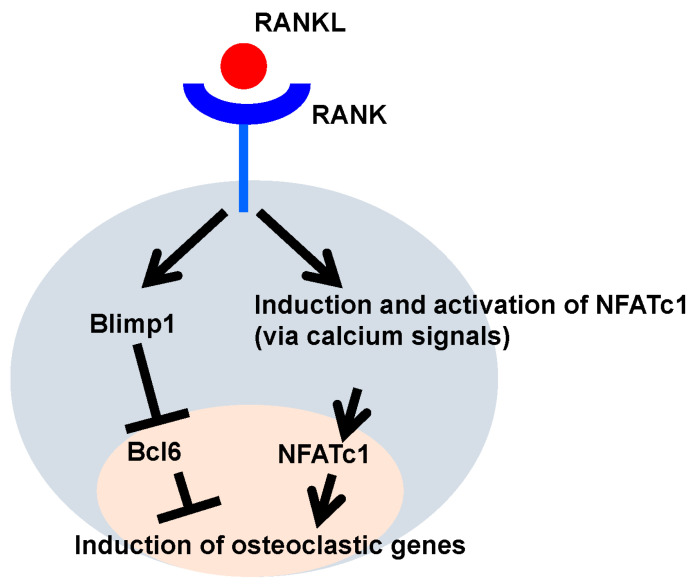
Regulation of osteoclastogenesis: NFATc1 and Blimp1-Bcl6 axis.

As described above, osteoclast differentiation is positively regulated downstream of RANKL by the transcription factor c-Fos-NFATc1 axis [20,21]. MAF bZIP transcription factor B (MafB), interferon regulatory factor 8 (Irf8), and B cell lymphoma 6 (Bcl6), all of which are transcriptional repressors, have also been identified as negative regulators of osteoclast differentiation [30,31,32]. As negative regulators of osteoclast differentiation, they play an inhibitory role in NFATc1, the accelerator of osteoclast differentiation. Mice deficient in either Irf8 or Bcl6 have been shown to exhibit a constant increase in osteoclast differentiation and activation, which in turn exhibits a decrease in bone mineral density [30,32]. The RANKL-RANK axis induces the expression and activation of NFATc1, a master transcription factor for osteoclast differentiation, via calcium signaling (Figure 1). However, NFATc1 expression and activation alone are not sufficient to promote osteoclast differentiation. Bcl6 is a transcriptional repressor that negatively regulates osteoclast differentiation (Figure 1). Indeed, the overexpression of Bcl6 inhibits osteoclast differentiation [32]. Meanwhile, B lymphocyte-induced maturation protein-1 (Blimp1) (also called PR domain containing 1, Prdm1), a transcriptional repressor downstream of RANKL, directly suppresses Bcl6 expression, resulting in inducing osteoclast differentiation (Figure 1) [32]. Blimp1 global knockout mice are embryonic lethal, but in conditional knockout mice lacking osteoclast-specific Blimp1, increased bone mass was observed due to inhibition of osteoclastogenesis [32,33]. Furthermore, Blimp1 conditional knockout mice, which are systemically deficient in Blimp1 after adulthood, also show an increase in bone mass due to the inhibition of osteoclastogenesis [34]. This suggests that Blimp1 may be a potential therapeutic target for increasing bone mass by inhibiting osteoclastogenesis [34].

## 5. Mechanisms Underlying Postmenopausal Osteoporosis

**Figure 2:**
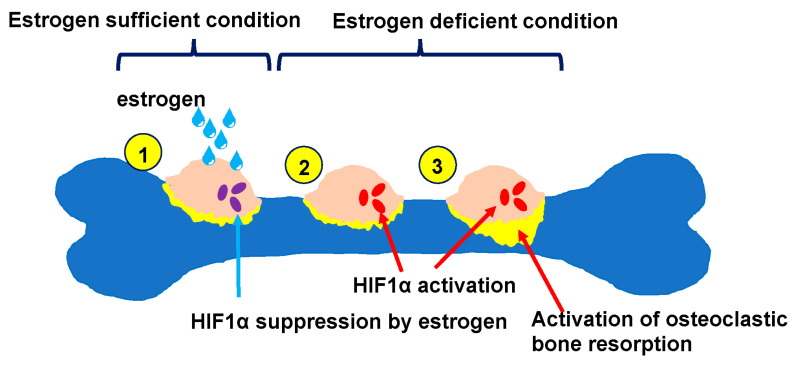
A mechanism underlying osteoclast activation in an estrogen-deficient condition. During the premenopausal estrogen sufficiency state, HIF1α, a transcriptional factor in osteoclasts, is continuously suppressed by estrogen (1). When estrogen deficiency occurs due to menopause, estrogen no longer suppresses HIF1α, and HIF1α is activated (2), which in turn the activation of osteoclasts leading to the development of osteoporosis (3).

In postmenopausal individuals, estrogen deficiency increases the activity of osteoclasts, which are responsible for bone resorption, leading to osteoporosis that is attributable to decreased bone mass. Moreover, menopause-related decreases in blood estrogen levels are known to increase levels of bone resorption markers in blood. Osteoclasts are found only on the bone’s surface in hypoxic regions marked by an extremely low oxygen concentration [35]. We have shown that osteoclasts express hypoxia inducible factor 1 alpha (HIF1α), a transcription factor that regulates responses to hypoxia, and that in the premenopausal estrogen-sufficiency state, HIF1α expression by osteoclasts is suppressed by estrogen in those hypoxic regions (Figure 2) [11]. The osteoclastic bone resorptive activity required for physiological bone remodeling is independent of HIF1α (Figure 2). However, in estrogen-deficient conditions following menopause onset, HIF1α expression and activity increases, promoting osteoclastic bone resorption and leading to osteoporosis development (Figure 2) [11]. In mice genetically engineered to lack HIF1α in osteoclasts, no bone loss occurs in a postmenopausal osteoporosis model (ovariectomy, OVX) in which both ovaries are removed [11]. Furthermore, after a screen for HIF1α-inhibiting agents, we identified a small molecule that efficiently inhibits HIF1α expression in osteoclasts [30]. When we administered it to OVX mice, bone loss caused by estrogen deficiency was completely inhibited [11]. We also found that testosterone inhibits HIF1α expression in osteoclasts [36]. We then found that, in a male osteoporosis model in which bilateral testes were removed (orchiectomy, ORX), the administration of our HIF1α inhibitor completely blocked bone loss in ORX mice as well [36]. We also demonstrated that the treatment of mice with either selective estrogen-receptor modulators (SERMs), such as raloxifene, bazedoxifene, or tamoxifen, or the activated vitamin D3 analogue eldecalcitol, inhibited HIF1α expression in osteoclasts [37,38,39].

## 6. Bone Remodeling

**Figure 3:**
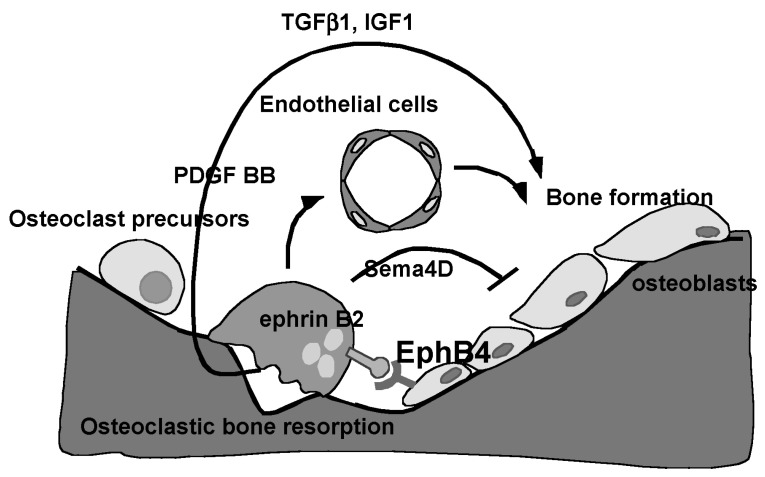
Regulation of bone remodeling. Bone remodeling is thought to be regulated by the activation of mesenchymal stem cells and osteoblasts by growth factors such as TGFβ1 and IGF1 released from the bone matrix by osteoclastic bone resorption. It has also been reported that ephrin B2, a membrane-bound ligand expressed by osteoclasts, stimulates Eph B4, a receptor expressed on osteoblasts. Semaphorin 4D (Sema 4D) secreted by osteoclasts inhibits bone formation, and PDGF BB secreted by osteoclasts promotes bone formation via angiogenesis.

Bone metabolism can be divided into two major types: bone remodeling, in which bone formation is activated after resorption, and bone modeling, in which bone is formed without resorption. Remodeling is seen in areas where osteoclasts are present, such as trabecular bone, while modeling is seen mainly in membranous ossification. Classically, the growth factors such as transforming growth factor beta 1 (TGFβ) and insulin-like growth factor 1 (IGF1), which accumulate in the bone matrix, are released and activated by osteoclast bone resorption, and in turn, stimulate bone remodeling by activating osteoblasts to stimulate bone formation (refs. [40,41], Figure 3). TGFβ is stored in the bone extracellular matrix protein in an inactive latent form (ref. [40], Figure 3), and latent-TGFβ is known to be converted to the active form by acid. During bone resorption, this acid is secreted by osteoclasts, and the active form of TGFβ acts as a remodeling factor that activates osteoblasts following bone resorption (ref. [40], Figure 3). However, some factors have been reported to act on the osteoblast side via factors expressed by differentiated osteoclasts (Figure 3) rather than on bone resorption (Figure 3). The concept of bone remodeling has diversified, as ephrin B2 expressed on osteoclasts stimulates Eph B4 expressed on osteoblasts to promote bone formation [42], semaphorin 4D (Sema4D) expressed on osteoclasts conversely inhibits bone formation [43], and PDGF BB expressed on osteoclasts promotes bone formation through angiogenesis [44]. The concept of bone remodeling has diversified (Figure 3).

## 7. Coupling and Uncoupling of Bone Metabolisms

In primary osteoporosis, a typical bone disease, both osteoclast and osteoblast activities are elevated, and when osteoclasts are suppressed with bisphosphonates or other bone resorption inhibitors, bone formation is also suppressed. Bone resorption inhibitors are currently the most commonly used drugs in the treatment of osteoporosis, as they suppress bone formation but increase bone mass by inhibiting excessive osteoclast activity. On the other hand, it has been suggested that the long-term administration of potent bone resorption inhibitors leads to the suppression of bone turnover, called severely suppressed bone turnover (SSBT), which may cause osteonecrosis of the jaw and atypical femur fractures [45,46,47,48]. Semaphorin 3a expressed by osteoblasts suppresses osteoclasts while activating osteoblasts, and it has been shown that both osteoclasts and osteoblasts can contribute to increased bone mass by creating an uncoupling state where the activities of the two cells are not synchronized [49]. Cathepsin K, a proteolytic enzyme that is activated under acidic conditions characteristic of osteoclasts and is used as a marker of osteoclast differentiation, has been reported to exhibit an uncoupling phenotype in an autosomal recessive genetic disease called pycnodysostosis and in gene-deficient mice, in which osteoclast activity increases rather than decreases [50,51]. A Cathepsin K inhibitor was previously developed as a therapeutic agent for increasing bone mass, although it was not launched due to an adverse effect. It has also been reported that the signal transducer and activator of transcription 1 (Stat1) acts to inhibit differentiation in both osteoclasts and osteoblasts, and although both cells are activated by Stat1 loss, osteoblast activation is relatively more dominant than osteoclast activation, resulting in increased bone mass [52,53]. Mutations in the SOST gene encoding the Sclerostin protein result in a disease that causes increased bone mass [54,55]. Similarly, mice lacking the SOST gene have an increased bone mass phenotype with elevated bone formation [56]. Treatment with Romosozumab, a neutralizing antibody against Sclerostin, increases bone mass by uncoupling the transient activation of osteoblasts with the sustained inhibition of osteoclast activity in osteoporosis patients [10]. Romosozumab treatment has been shown to significantly prevent fragility fractures as a treatment for osteoporosis [57].

## 8. Treatment Strategy for Osteoporosis

Osteoporosis drugs increase bone mineral density (BMD) and reduce fragility fractures by intervening in bone metabolism. In women aged 55–81 years, alendronate, which is a bisphosphonate, significantly reduced the incidence of clinical vertebral fractures (hazard reduction 55%, *p* < 0.001) compared with a placebo for 3 years of treatment, and also significantly reduced hip fractures, which are difficult to prevent (relative hazard 0.49, *p* = 0.047). [58]. Denosumab, a neutralizing antibody against RANKL, significantly reduced the risk of new vertebral fracture (hazard ratio, 0.32; *p* < 0.001) and hip fracture (hazard ratio, 0.60; *p* = 0.04) in osteoporotic women aged 60 to 90 years after 3 years of treatment with denosumab versus placebo (hazard ratio, 0.60; *p* = 0.04). [9]. Teriparatide (PTH 1–34) has been shown to significantly reduce the risk of vertebral fracture versus placebo in postmenopausal osteoporotic patients treated for an average of 21 months (relative risk 0.35: *p* < 0.001). [59].

A potential limitation of osteoporosis drugs is that some have a limited duration of administration and others do not, making it difficult to compare long-term outcomes for differences in efficacy between these drugs. In postmenopausal women with low bone mineral density, romosozumab was superior to alendronate, a bisphosphonate, and teriparatide, an osteogenic agent, at 6 and 12 months after treatment, and BMD has been shown to increase significantly in the lumbar spine, proximal femur, and femoral neck at 6 and 12 months after treatment with romosozumab. [10]. Abaloparatide and teriparatide, both of which are bone-forming agents, also significantly reduced the incidences of new morphometric vertebral fractures in postmenopausal women with osteoporosis in both groups, as compared to the placebo (Abaloparatide group: relative risk, 0.14 [95% CI, 0.05 to 0.39]; *p* < 0.001, teriparatide group: relative risk, 0.20 [95% CI, 0.08 to 0.47]; *p* < 0.001), but there was no significant difference between the two groups [60].

On the other hand, the fact that the majority of hip fracture patients were not receiving treatment for osteoporosis at the time of fracture [61] makes it more important to ensure that drugs reach patients who need treatment than to develop drugs with high efficacy.

## 9. Joint Erosion and Osteoclasts in RA

In RA, in addition to the chronic inflammation of the joints, the activation of osteoclasts has been known to occur locally in the joints. In addition, when the types of T cell subsets were still poorly known, arthritis was thought to be caused by the activity of TH1 cells, which induce inflammation. However, because TH1 cells produce interferon gamma (IFNγ), a strong osteoclast suppressor, the activation of osteoclasts in a TH1-activated environment is inconsistent. TH17 cells, which produced IL-17 but not IFNγ, have been identified, and IL-17-deficient mice have been shown to exhibit improved pathogenesis of arthritis [62,63]. The TH17 cell is now considered to be an essential component in the pathogenesis of arthritis. Nowadays, single RNA sequencing or genome-wide studies using RA patient samples are being used to further elucidate the pathogenesis of the disease [64,65].

Osteoclasts play a major role in joint erosion and destruction in RA patients. This evidence has been shown in several osteoclast-deficient mouse models of arthritis. It has been reported that joint destruction in the human TNFα transgenic (hTNF Tg) was reportedly abolished by crossing it with c-Fos-deficient mice, in which osteoclasts were completely absent [66]. However, joint inflammation in the hTNF Tg/c-Fos-deficient mice was also observed in hTNF Tg mice, suggesting a distinction between arthritis and joint destruction [66]. In addition, an adjuvant-induced arthritis model in rats has shown that osteoprotegerin (OPG) administration can inhibit bone and cartilage destruction but not arthritis [67]. These findings indicate that joint destruction is caused by osteoclasts and that joint destruction can be prevented if osteoclasts can be suppressed even if inflammation remains severe. In RA patients, the administration of denosumab, a RANKL-neutralizing antibody, has been shown to significantly inhibit joint erosion by inhibiting osteoclast activity but not the joint-space narrowing caused by arthritis.

## 10. Chronic Inflammation in Rheumatoid Arthritis and Osteoporosis

**Figure 4:**
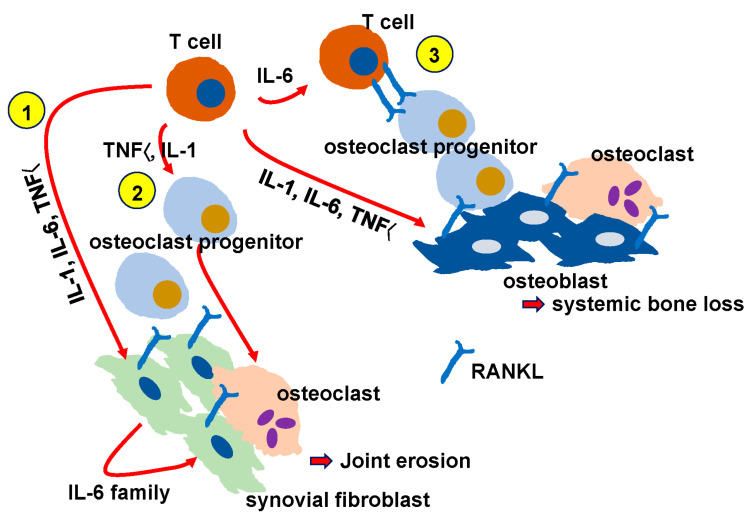
Inflammatory cytokine network for joint erosion and systemic bone loss in RA. T cells activated by antigen presentation, such as citrullinated proteins, secrete inflammatory cytokines such as TNFα, IL-6, and IL-1 (1). All of these inflammatory cytokines act on synovial fibroblasts to activate the expression of inflammatory cytokines such as the IL-6 family. These inflammatory cytokines also induce osteoclast differentiation by inducing RANKL expression in synovial fibroblasts (1). TNFα directly activates osteoclast differentiation, while IL-1 prolongs osteoclast survival (2). IL-6 also activates T cells and induces osteoclast differentiation by inducing RANKL expression (3).

**Figure 5:**
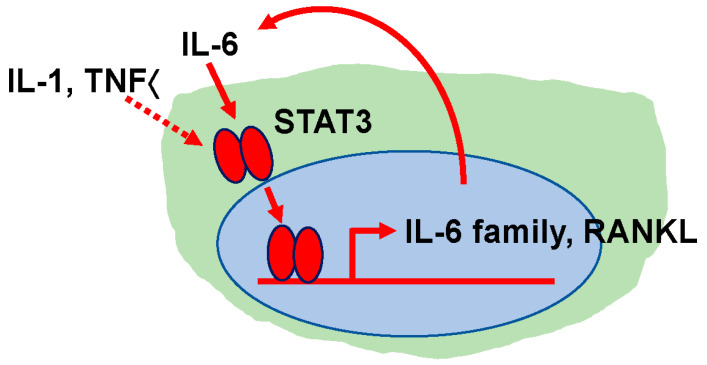
A positive feedback loop of inflammatory cytokine and RANKL expression. IL-6 directly activates and TNFα and IL-1 indirectly activate the transcription factor STAT3. The activated STAT3 induces the expression of inflammatory cytokines such as the IL-6 family, forming a positive feedback loop in which inflammatory cytokines are induced by inflammatory cytokines. The IL-6 family induced by the STAT3 activation further activates STAT3, thereby continuing this loop. The activated STAT3 also induces RANKL expression and activates osteoclast differentiation. This RANKL expression is also continued in a positive feedback loop by inflammatory cytokines.

In patients with RA, blood or local concentrations of inflammatory cytokines such as TNFα, IL-1, and IL-6 are generally elevated, particularly in older patients, causing joint swelling and destruction [68] via osteoclast activation. These factors are expressed by synovial fibroblasts and inflammatory cells infiltrating the joint synovium at the site of inflammation, and their concentration increases at the joint site, increasing osteoclast activity at that site and promoting pannus formation, joint destruction, and joint deformation (Figure 4) [69]. Together or alone, TNFα, IL-1 or IL-6 stimulate the expression of inflammatory cytokines, such as other IL-6 family proteins in synovial fibroblasts, thus promoting inflammatory cytokine expression in a positive feedback manner (Figure 4-1 and Figure 5) [69]. This activity further promotes the expression of RANKL, which is essential for osteoclast differentiation, induction, and activation, to induce osteoclastogenesis (Figure 4-1 and Figure 5) [69]. TNFα reportedly induces osteoclast activity directly (Figure 4-2) [70]. RANKL is a member of the TNFα superfamily, and some evidence suggests that intracellular signaling via RANKL or TNFα is similar. The TNF receptor-associated factor 6 (TRAF6) was demonstrated to be required for RANKL-induced osteoclastogenesis [71,72]. On the other hand, RANKL is required for osteoclast differentiation, while TNFα reportedly promotes the activation of osteoclast progenitor cells [73]. IL-1 is known to act directly on osteoclasts or osteoclast progenitors (Figure 4-2) [74], causing osteoclast activation and the prolonged survival of osteoclasts. Furthermore, TNFα, IL-1, and IL-6 induce RANKL expression by acting on osteoblasts (Figure 4) [69], thereby contributing to osteoclast induction. IL-6 also reportedly contributes to RANKL expression in T cells and osteoclast induction in synovial cells in joints (Figure 4-3) [75]. It is also possible that inflammatory cytokines expressed in joint tissues may increase IL-6 levels in the blood, resulting in a systemic increase in osteoclast activity. TNFα, IL-1, and IL-6 also activate signal transducer and activator of transcription factor 3 (STAT3), either directly (via IL-6) or indirectly (via TNFα and IL-1), and activated STAT3 promotes the expression of the IL-6 family cytokines and RANKL in fibroblasts (Figure 5) [69]. IL-6 family cytokines induced by activated STAT3 are known to enhance the expression of IL-6 family cytokines and RANKL, furthering inflammation and joint destruction (Figure 5) [69]. The induced IL-6 family cytokines further activate STAT3 to continuously induce IL-6 family cytokines and RANKL (Figure 5). We found that positive feedback loops of proinflammatory cytokines are not triggered by proinflammatory cytokines in Stat3-deficient cells, indicating that Stat3 induces the triggering of positive feedback loops of proinflammatory cytokines [69]. In Stat3-deficient cells, the induction of RANKL expression by inflammatory cytokines was also inhibited [69]. Furthermore, the conditional deletion of Stat3 at an adult stage was demonstrated to significantly inhibit arthritis development in RA mouse models [76]. RA susceptibility is also a risk factor for osteoporosis and fragility fractures, as RA is listed as a risk for osteoporosis in the Fracture Risk Assessment tool (FRAX) used to assess the risk of fragility fractures due to osteoporosis.

By contrast, elevated levels of inflammatory cytokines contribute to bone loss not only by activating osteoclasts but by suppressing bone formation by inhibiting osteoblast activity [77]. Some reports state that in RA patients, joint erosion can be rescued by blocking inflammation with biologics [78]. In RA patients, the induction of factors that suppress the Wnt-β catenin signaling that promotes bone formation is also thought to contribute to bone loss [79,80].

## 11. Either Methotrexate (MTX) or CTLA4-Ig Directly Inhibit Osteoclast Differentiation

Methotrexate (MTX) is the anchor drug for RA therapy and is the first-line agent for patients with RA who are eligible for MTX. MTX was originally developed as an anticancer drug and was probably first administered to RA patients to suppress proliferating synovial cells. In fact, however, it has been shown to cause marked improvement of joint swelling and joint tenderness in RA patients [81]. We found that MTX significantly inhibited osteoclast differentiation in osteoclast cultures in vitro, and that MTX significantly inhibited calcium signaling and NFATC1 expression in the osteoclast progenitor cells induced by RANKL [82]. NFAT is also essential for T cell activation, and stimulation from the T cell receptor (TCR) activates NFAT. MTX is considered to exert its osteoclast-differentiation inhibitory and immunosuppressive effects by suppressing NFAT in osteoclasts and T cells, respectively.

Similarly, we demonstrated that CTLA4-Ig, which suppresses co-stimulatory signaling, also significantly inhibited osteoclast differentiation in vitro [83]. We showed that CTLA4-Ig significantly inhibited calcium signaling and NFATC1 expression in osteoclast progenitor cells induced by RANKL via FcRγ, an ITAM signaling molecule [83]. Thus, CTLA4-Ig also directly inhibits both osteoclasts and T cells, and it may exert joint-erosion inhibitory and immunosuppressive activity.

## 12. Glucocorticoid-Induced Osteoporosis

Although glucocorticoid treatment has long been essential in treating RA, particularly in patients who cannot tolerate MTX [84], it also could induce secondary osteoporosis [85]. Glucocorticoid administration induces bone loss by transiently activating osteoclasts early in the course of treatment, which thereby inhibits bone formation in mid- to long-term treatment stages. Glucocorticoid administration also reportedly increases the risk of fractures at various sites throughout the body [86,87], an effect more pronounced in women than in men [86]. Patients with RA who are treated with glucocorticoids reportedly have a higher risk of fracture than those not treated with glucocorticoids [88]. On the other hand, fracture risk gradually decreases when glucocorticoid administration is discontinued, often disappearing about a year later [86]. Fracture risk with glucocorticoid administration is also dose-dependent, with prednisolone doses equivalent to 7.5 mg/day or higher greatly increasing fracture risk [86]. Increased age and the presence of preexisting fractures also increase fracture risk with glucocorticoid administration [88]. On the other hand, high lumbar bone density and treatment with bisphosphonates can reduce fracture risk [85]. In various countries, approaches have been proposed to assess risk based on age, bone density, existing fractures, and glucocorticoid dosage and to recommend medication for fracture prevention.

## 13. Aging of RA Patients and Risk of Osteoporosis

Interestingly, the age of RA onset has increased in recent years, although the causes are not well understood [89]. Potentially relevant to this observation is the fact that RA treatment advances have improved the prognosis of RA patients. Although not limited to RA patients, it is well known that aging is a risk factor for osteoporosis in general, and its prevalence increases with age [2]. Osteoporosis is a risk for fragility fractures; therefore, the occurrence of fragility fractures should be noted in RA patients. Osteoporosis is also associated with sarcopenia [90,91,92]; thus, one should be aware of events such as falls that are related to the development of fragility fractures.

Among fragility fractures caused by osteoporosis, hip fractures are the most serious. According to a survey of conducted by Kumamoto University Hospital, the average age of hip fracture patients is approximately 85 years [61]. In addition, most of the patients enrolled in the hip fracture study had hip fractures due to falls. In our study, decreased grip strength, a diagnostic criterion for sarcopenia, was also identified as a factor significantly associated with hip fracture (Table 1 and Table 2) [61]. Thus, in the condition known as rheumatoid sarcopenia [93], attention should be paid to the occurrence of fragility fractures such as hip fractures due to falls.

## 14. Conclusive Remarks

The main focus of the present article is to introduce the mechanisms of osteoporosis development and bone erosion rather than to offer a systematic review or statement. Therefore, rather than presenting an introduction to the literature with an aligned methodology and population, as in a systematic review, the papers were selected to highlight why an understanding of the mechanism is necessary. Thus, there is the possibility of concerns about the limitations of this manuscript, including the potential risk of bias in the included studies, gaps in the evidence base due to the lack of condition-based selection from an extensive article search, and differences in methodology and population among the cited papers. In any case, this paper provides a comprehensive overview of the pathogenesis and regulation of osteoporosis, fragility fractures, and bone erosion in rheumatoid arthritis from various perspectives. RA and osteoporosis are closely related, and RA treatment requires constant vigilance to monitor patients for the development of osteoporosis. While both patients and physicians are very aware of joint pain, erosion, destruction, and deformity, they tend to be less aware of osteoporosis, which has fewer symptoms. In this article, we have introduced three key osteoporosis risk factors in the context of RA: inflammatory disease, glucocorticoid administration, and advanced age. In particular, elderly patients with RA often experience all three factors and require special attention. In any case, bone mineral density examination, checking for preexisting fractures, or utilizing FRAX are actionable strategies for assessing osteoporosis development in RA patients and should be performed as part of routine care. Basic science is an extremely useful tool for elucidating the molecular mechanisms underlying the regulation of bone homeostasis and the pathogenesis of bone erosion in RA patients, allowing us to do things that cannot be performed in patients, such as creating knockout mice that target specific molecules and then extrapolating the results to a clinical setting. In fact, basic science, including understanding molecular mechanisms, has led to the development of therapeutic agents such as denosumab. In RA and in osteoporosis, sarcopenia is of particular importance, and patients with RA who develop osteoporosis should be treated to prevent fall fractures due to rheumatoid sarcopenia. If patients are scheduled to receive oral glucocorticoid treatment for at least 3 months, they could be prescribed anti-osteoporosis drugs prophylactically if they will receive at least 7.5 mg/day of prednisolone, have an existing fracture, or are 65 or older. Overall, in this era, awareness of RA is required to protect patients from bone fractures.

## 15. Future Direction of Osteoporosis and Bone Erosion Management

The number of patients with osteoporosis is expected to continue to increase with the growth of the elderly population, and the number of patients with fragility fractures such as hip fractures is also expected to increase. More detailed bone evaluation may be possible currently or in the near future. HR-pQCT is useful in the evaluation of bone microstructure and is able to show in detail the differences in bone responses to different osteoporosis drugs in postmenopausal osteoporosis patients [94]. In RA patients, HR-pQCT can be a useful tool in the evaluation of bone erosion because it can target the small joints and metacarpals of the fingers [95,96]. However, there is currently a limit to the size of bone that can be analyzed with HR-pQCT, and the femur and lumbar spine cannot be analyzed. Evaluation of regional volumetric BMD and biomechanical parameters using the Quantitative Computed Tomography (QCT)-based Finite Element Method (FEM) has also been proposed [97]. In addition, although ultrasonography has been thought to be unsuitable for bone mass assessment, BMD assessment of the lumbar spine and femur using ultrasonography has been proposed, and consistency with BMD measured by DXA has been demonstrated [98]. In the future, it is expected to be a modality without X-Ray exposure, such as DXA, HR-pQCT, and QCT-based FEM. Neither modality is yet at a stage where it can be used for a large number of patients in terms of widespread use.

With the widespread use of detailed bone erosion assessment like HR-pQCT, it may become clinically feasible to quantify the volume of bone erosion and its changes during the treatment. The incorporation of AI methods such as machine learning may make it possible to automate the assessment of Sharp scores and joint space narrowing and their changes in a timely manner and reduce the time required for these measurements in the future. Accumulation of data from longitudinal studies and the long-term follow-up of changes in joint statuses in RA patients would also be useful in future studies to evaluate in detail the effect of drugs in preventing joint destruction. In addition, basic science is still considered necessary. If the etiology rather than the pathogenesis of RA is clarified, the subgrouping of RA patients and more fundamental treatment may become possible in the future.

Because osteoporosis is associated with sarcopenia and fragility fractures [90,92], it is necessary to provide total care for these conditions in the elderly. A total of 93% of hip fracture patients were not receiving treatment for osteoporosis at the time of fracture [61]. If individuals have RA or other diseases that cause osteoporosis, they should receive examination and treatment for osteoporosis in conjunction with treatment for the particular diseases. In addition, the average age of hip fracture patients is approximately 85 years old [61], and thus, it is necessary to anticipate and prevent future osteoporosis and fragility fractures earlier rather than taking action at age 85. Toward this end, it will be necessary to improve the accuracy of prediction tools for future osteoporosis and fragility fractures [91]. A longitudinal study will recommend whether intervening before fracture can prevent fracture by identifying older adults who are at high risk.

## Figures and Tables

**Table 1:** Scores for the categories of each risk factor including items that need to be measured in the hospital.

Features	Features Importance	Score ^a^
Serum 25OHD (ng/mL)		
10≥		0
10<	0.659	7
Femoral neck T-score		
≥−3.0		0
<−3.0	0.459	5
Total Barthel index score		
100		0
<100	0.349	3
Maximal handgrip strength (kg)		
18≥		0
18<	0.334	3
Locomotive syndrome		
<24		0
≥24	0.218	2
History of falls within 1 year		
<2		0
≥3	0.209	2
sIGF-1		
≥50		0
<50	0.189	2
Tea (cups/day)		
≤4		0
=5	−0.189	−2 ^b^
Osteoporosis drugs		
No		0
Yes	−0.179	−1 ^b^
Body mass index		
≥18.5		0
<18.5	0.070	1

^a^ Calculated 10 times for each parameter estimate and decimals rounded off. ^b^ The scores of the protective factors (tea and osteoporosis drug) were expressed by negative numbers.

**Table 2:** Scores for the categories of each risk factor not including items that need to be measured in the hospital.

Features	Features Importance	Score ^a^
Total Barthel index score		
100		0
<100	0.505	5
Locomotive syndrome		
<24		0
≥24	0.433	4
History of falls within 1 year		
<2		0
≥3	0.243	2
Tea (cups/day)		
≤4		0
=5	−0.231	−2 ^b^
Cognitive impairment		
None		0
Yes	0.161	2
Osteoporosis drugs		
No		0
Yes	−0.133	−1 ^b^
Use of walking aid		
Yes		0
No	0.094	1
Walking habit		
No		0
Yes	0.053	1

^a^ Calculated 8 times for each parameter estimate and decimals rounded off. ^b^ The scores of protective factors (tea and osteoporosis drug) were expressed by negative numbers.
